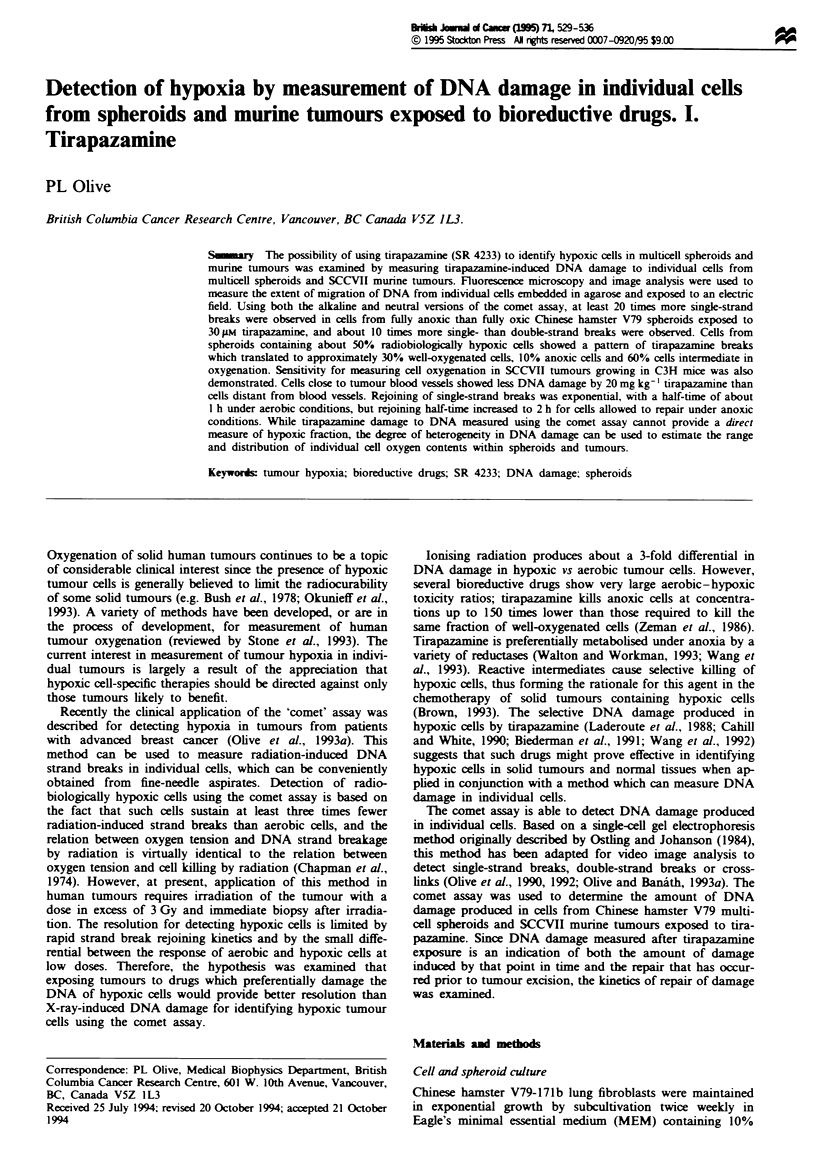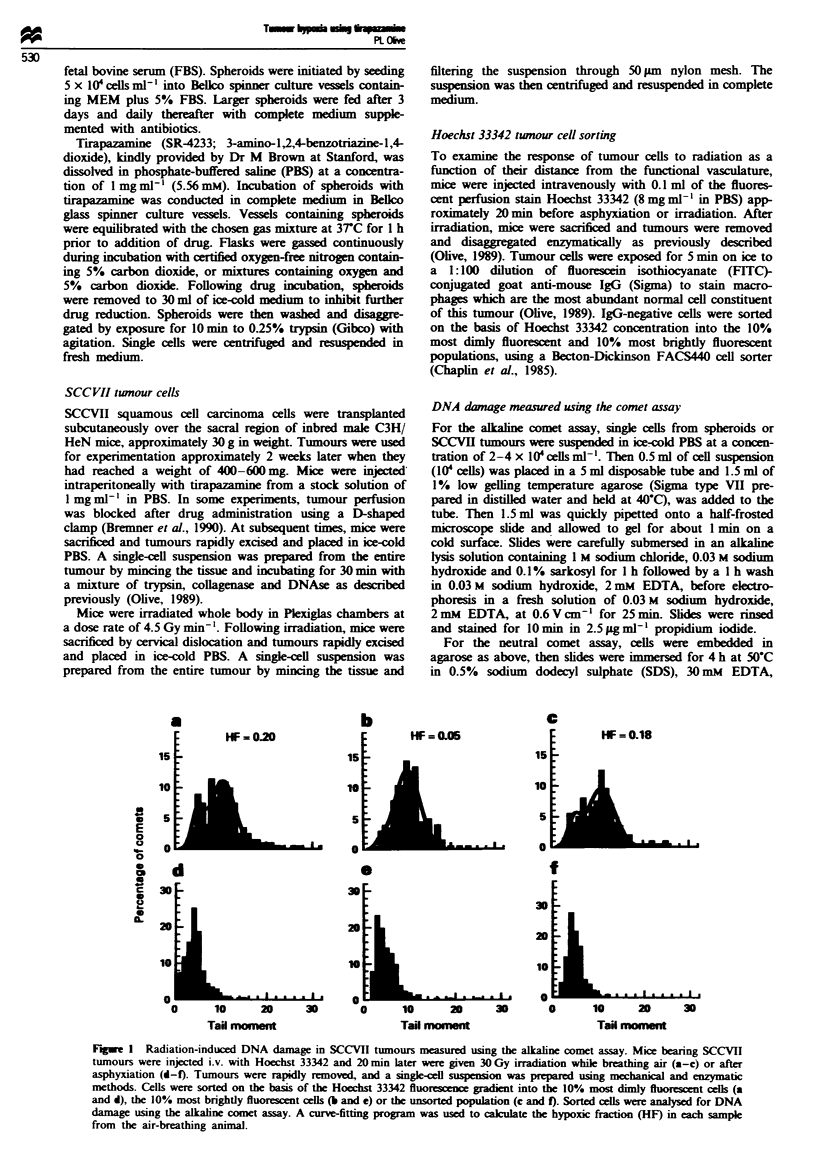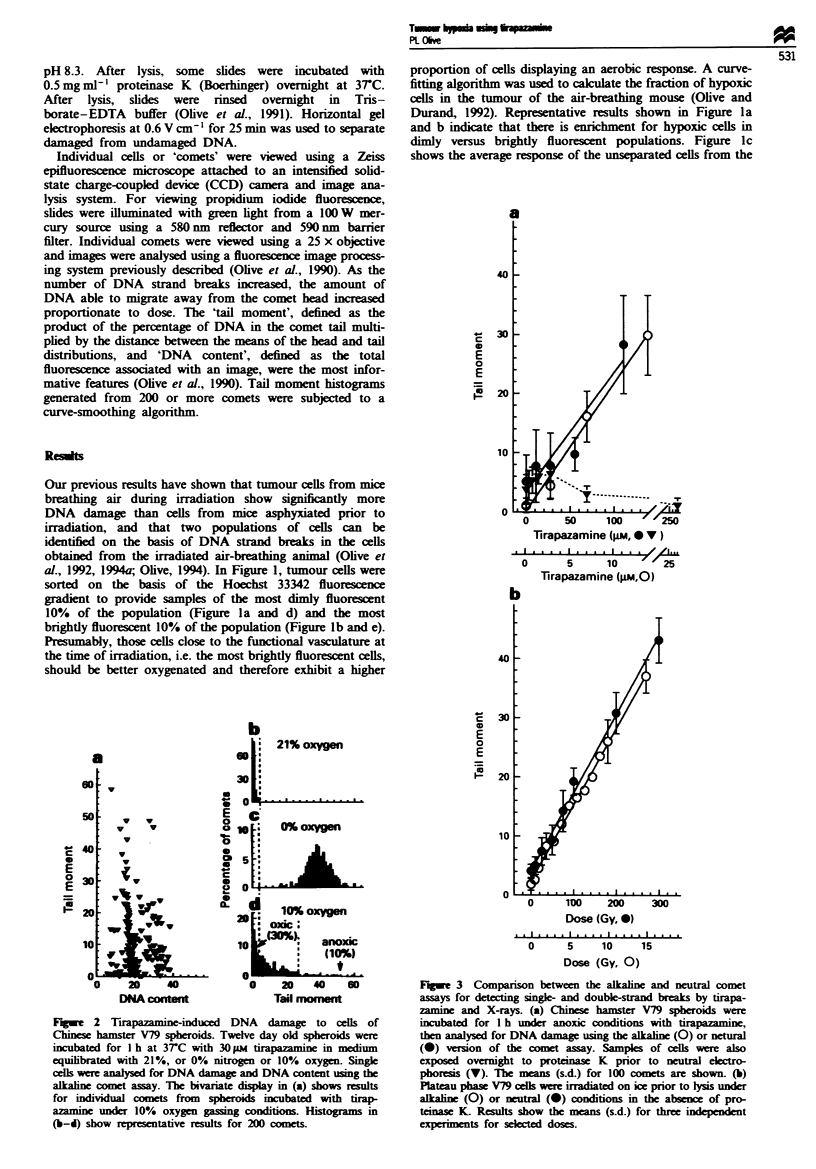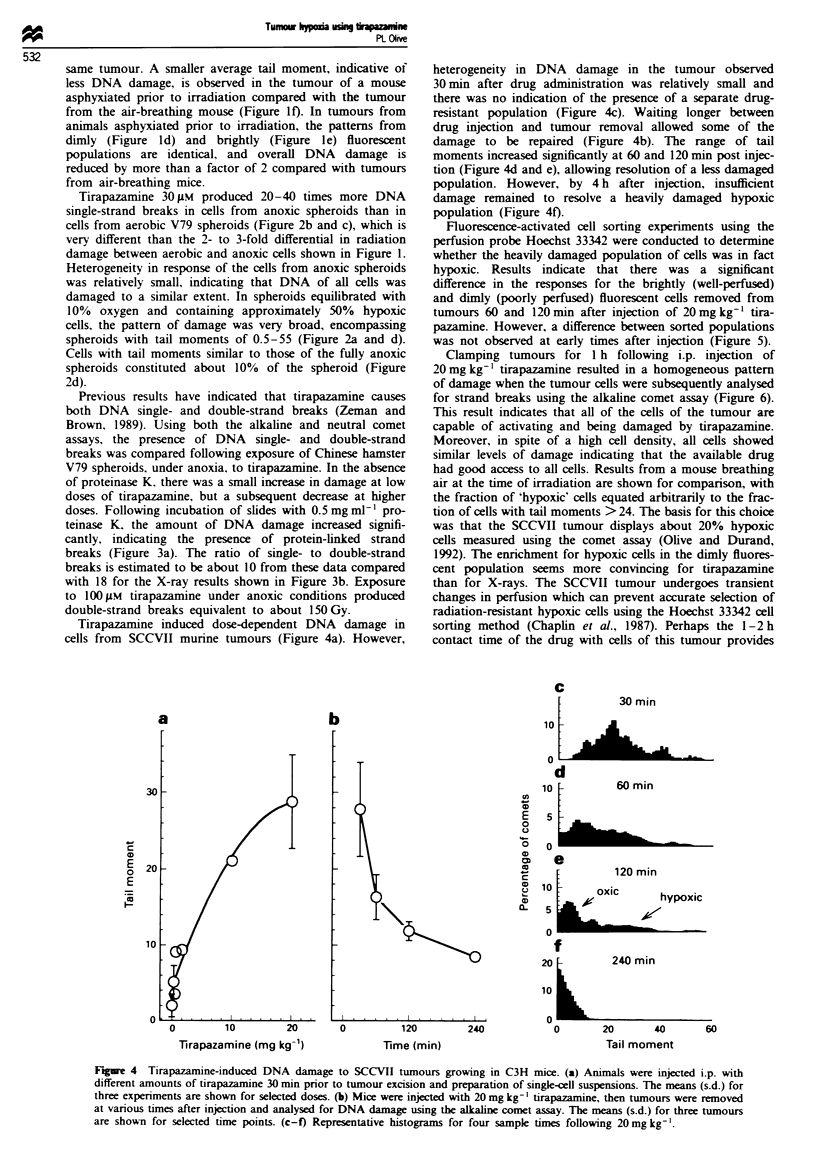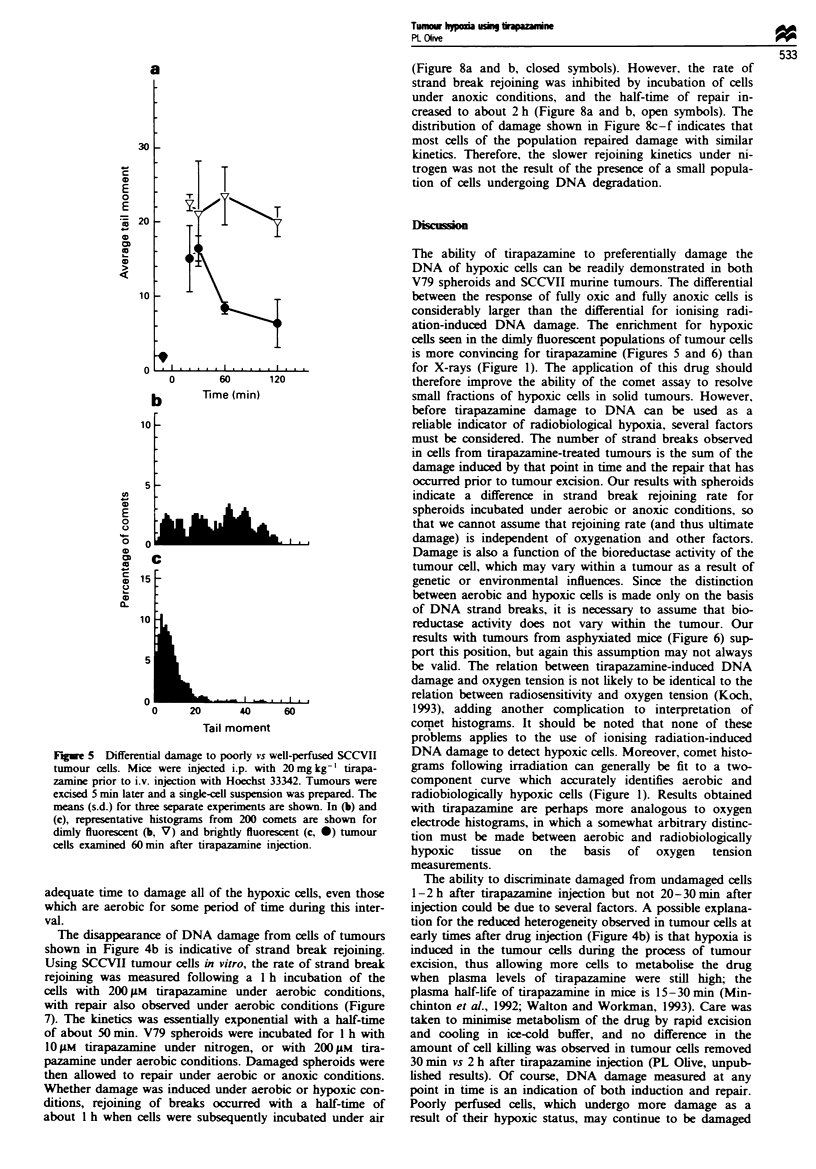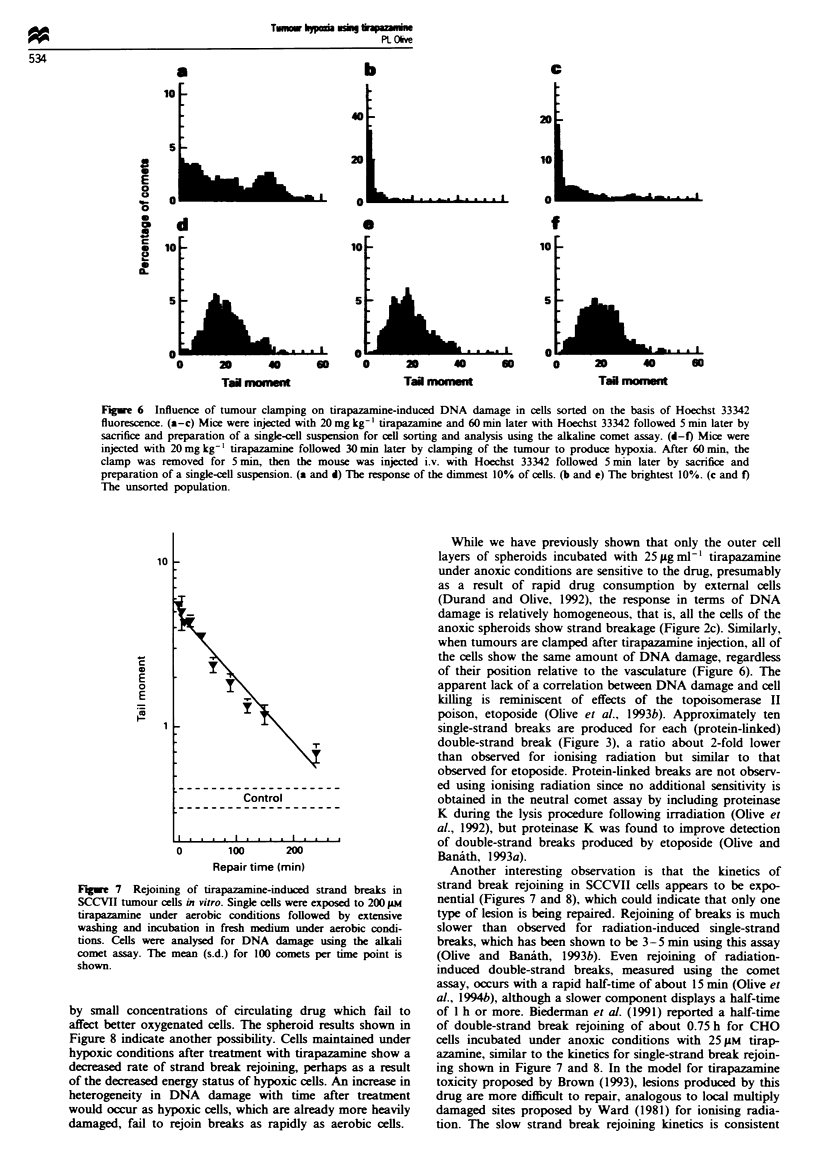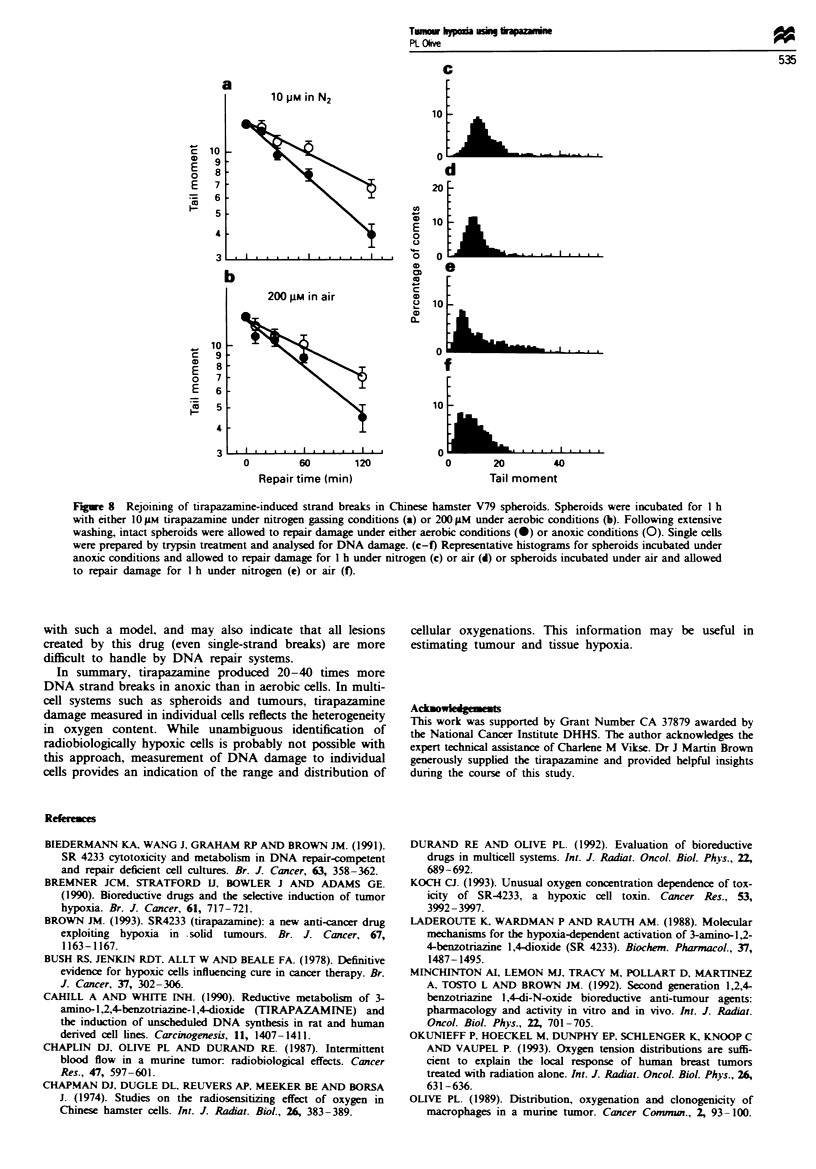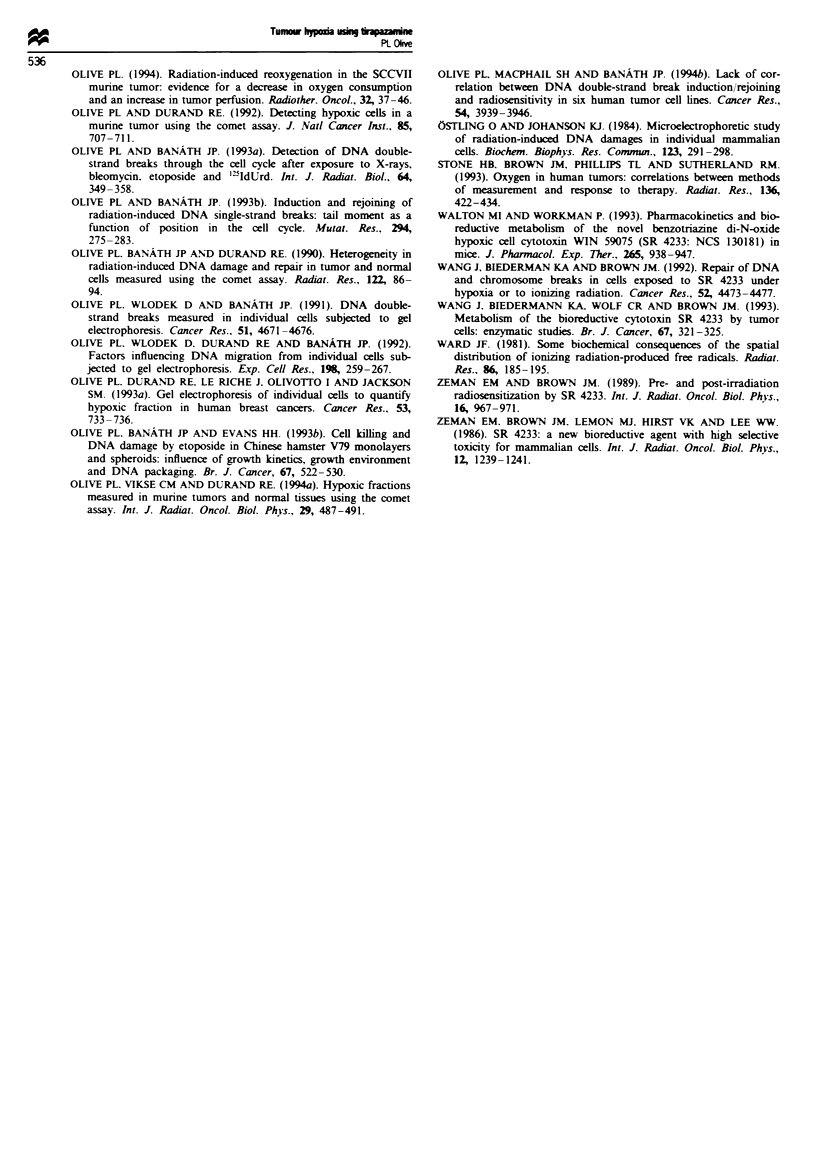# Detection of hypoxia by measurement of DNA damage in individual cells from spheroids and murine tumours exposed to bioreductive drugs. I. Tirapazamine.

**DOI:** 10.1038/bjc.1995.105

**Published:** 1995-03

**Authors:** P. L. Olive

**Affiliations:** British Columbia Cancer Research Centre, Vancouver, Canada.

## Abstract

The possibility of using tirapazamine (SR 4233) to identify hypoxic cells in multicell spheroids and murine tumours was examined by measuring tirapazamine-induced DNA damage to individual cells from multicell spheroids and SCCVII murine tumours. Fluorescence microscopy and image analysis were used to measure the extent of migration of DNA from individual cells embedded in agarose and exposed to an electric field. Using both the alkaline and neutral versions of the comet assay, at least 20 times more single-strand breaks were observed in cells from fully anoxic than fully oxic Chinese hamster V79 spheroids exposed to 30 microM tirapazamine, and about 10 times more single- than double-strand breaks were observed. Cells from spheroids containing about 50% radiobiologically hypoxic cells showed a pattern of tirapazamine breaks which translated to approximately 30% well-oxygenated in SCCVII tumors growing in C3H mice was also demonstrated. Cells close to tumour blood vessels showed less DNA damage by 20 mg kg-1 tirapazamine than cells distant from blood vessels. Rejoining of single-strand breaks was exponential, with a half-time of about 1 h under aerobic conditions, but rejoining half-time increased to 2 h for cells allowed to repair under anoxic conditions. While tirapazamine damage to DNA measured using the comet assay cannot provide a direct measure of hypoxic fraction, the degree of heterogeneity in DNA damage can be used to estimate the range and distribution of individual cell oxygen contents within spheroids and tumours.


© 1995 Stockton Press All rights reserved 0007-0920/95 $9.00

### Detection of hypoxia by measurement of DNA damage in individual cells from spheroids and murine tumours exposed to bioreductive drugs. I. Tirapazamine

PL Olive

*British Columbia Cancer Research Centre, Vancouver, BC Canada V5Z 1L3.*

**Summary** The possibility of using tirapazamine (SR 4233) to identify hypoxic cells in multicell spheroids and murine tumours was examined by measuring tirapazamine-induced DNA damage to individual cells from multicell spheroids and SCCVII murine tumours. Fluorescence microscopy and image analysis were used to measure the extent of migration of DNA from individual cells embedded in agarose and exposed to an electric field. Using both the alkaline and neutral versions of the comet assay, at least 20 times more single-strand breaks were observed in cells from fully anoxic than fully oxic Chinese hamster V79 spheroids exposed to 30 μM tirapazamine, and about 10 times more single- than double-strand breaks were observed. Cells from spheroids containing about 50% radiobiologically hypoxic cells showed a pattern of tirapazamine breaks which translated to approximately 30% well-oxygenated cells, 10% anoxic cells and 60% cells intermediate in oxygenation. Sensitivity for measuring cell oxygenation in SCCVII tumours growing in C3H mice was also demonstrated. Cells close to tumour blood vessels showed less DNA damage by 20 mg $kg^{-1}$ tirapazamine than cells distant from blood vessels. Rejoining of single-strand breaks was exponential, with a half-time of about 1 h under aerobic conditions, but rejoining half-time increased to 2 h for cells allowed to repair under anoxic conditions. While tirapazamine damage to DNA measured using the comet assay cannot provide a *direct* measure of hypoxic fraction, the degree of heterogeneity in DNA damage can be used to estimate the range and distribution of individual cell oxygen contents within spheroids and tumours.

**Keywords:** tumour hypoxia; bioreductive drugs; SR 4233; DNA damage; spheroids

Oxygenation of solid human tumours continues to be a topic of considerable clinical interest since the presence of hypoxic tumour cells is generally believed to limit the radiocurability of some solid tumours (e.g. Bush *et al.*, 1978; Okunieff *et al.*, 1993). A variety of methods have been developed, or are in the process of development, for measurement of human tumour oxygenation (reviewed by Stone *et al.*, 1993). The current interest in measurement of tumour hypoxia in individual tumours is largely a result of the appreciation that hypoxic cell-specific therapies should be directed against only those tumours likely to benefit.

Recently the clinical application of the 'comet' assay was described for detecting hypoxia in tumours from patients with advanced breast cancer (Olive *et al.*, 1993*a*). This method can be used to measure radiation-induced DNA strand breaks in individual cells, which can be conveniently obtained from fine-needle aspirates. Detection of radiobiologically hypoxic cells using the comet assay is based on the fact that such cells sustain at least three times fewer radiation-induced strand breaks than aerobic cells, and the relation between oxygen tension and DNA strand breakage by radiation is virtually identical to the relation between oxygen tension and cell killing by radiation (Chapman *et al.*, 1974). However, at present, application of this method in human tumours requires irradiation of the tumour with a dose in excess of 3 Gy and immediate biopsy after irradiation. The resolution for detecting hypoxic cells is limited by rapid strand break rejoining kinetics and by the small differential between the response of aerobic and hypoxic cells at low doses. Therefore, the hypothesis was examined that exposing tumours to drugs which preferentially damage the DNA of hypoxic cells would provide better resolution than X-ray-induced DNA damage for identifying hypoxic tumour cells using the comet assay.

Ionising radiation produces about a 3-fold differential in DNA damage in hypoxic *vs* aerobic tumour cells. However, several bioreductive drugs show very large aerobic–hypoxic toxicity ratios; tirapazamine kills anoxic cells at concentrations up to 150 times lower than those required to kill the same fraction of well-oxygenated cells (Zeman *et al.*, 1986). Tirapazamine is preferentially metabolised under anoxia by a variety of reductases (Walton and Workman, 1993; Wang *et al.*, 1993). Reactive intermediates cause selective killing of hypoxic cells, thus forming the rationale for this agent in the chemotherapy of solid tumours containing hypoxic cells (Brown, 1993). The selective DNA damage produced in hypoxic cells by tirapazamine (Laderoute *et al.*, 1988; Cahill and White, 1990; Biederman *et al.*, 1991; Wang *et al.*, 1992) suggests that such drugs might prove effective in identifying hypoxic cells in solid tumours and normal tissues when applied in conjunction with a method which can measure DNA damage in individual cells.

The comet assay is able to detect DNA damage produced in individual cells. Based on a single-cell gel electrophoresis method originally described by Ostling and Johanson (1984), this method has been adapted for video image analysis to detect single-strand breaks, double-strand breaks or cross-links (Olive *et al.*, 1990, 1992; Olive and Banáth, 1993*a*). The comet assay was used to determine the amount of DNA damage produced in cells from Chinese hamster V79 multicell spheroids and SCCVII murine tumours exposed to tirapazamine. Since DNA damage measured after tirapazamine exposure is an indication of both the amount of damage induced by that point in time and the repair that has occurred prior to tumour excision, the kinetics of repair of damage was examined.

### Materials and methods

### *Cell and spheroid culture*

Chinese hamster V79-171b lung fibroblasts were maintained in exponential growth by subcultivation twice weekly in Eagle's minimal essential medium (MEM) containing 10%

Correspondence: PL Olive, Medical Biophysics Department, British Columbia Cancer Research Centre, 601 W. 10th Avenue, Vancouver, BC, Canada V5Z 1L3
Received 25 July 1994; revised 20 October 1994; accepted 21 October 1994

fetal bovine serum (FBS). Spheroids were initiated by seeding $5 \times 10^4$ cells $ml^{-1}$ into Bellco spinner culture vessels containing MEM plus 5% FBS. Larger spheroids were fed after 3 days and daily thereafter with complete medium supplemented with antibiotics.

Tirapazamine (SR-4233; 3-amino-1,2,4-benzotriazine-1,4-dioxide), kindly provided by Dr M Brown at Stanford, was dissolved in phosphate-buffered saline (PBS) at a concentration of 1 mg $ml^{-1}$ (5.56 mM). Incubation of spheroids with tirapazamine was conducted in complete medium in Bellco glass spinner culture vessels. Vessels containing spheroids were equilibrated with the chosen gas mixture at 37°C for 1 h prior to addition of drug. Flasks were gassed continuously during incubation with certified oxygen-free nitrogen containing 5% carbon dioxide, or mixtures containing oxygen and 5% carbon dioxide. Following drug incubation, spheroids were removed to 30 ml of ice-cold medium to inhibit further drug reduction. Spheroids were then washed and disaggregated by exposure for 10 min to 0.25% trypsin (Gibco) with agitation. Single cells were centrifuged and resuspended in fresh medium.

*SCCVII tumour cells*

SCCVII squamous cell carcinoma cells were transplanted subcutaneously over the sacral region of inbred male C3H/HeN mice, approximately 30 g in weight. Tumours were used for experimentation approximately 2 weeks later when they had reached a weight of 400–600 mg. Mice were injected intraperitoneally with tirapazamine from a stock solution of 1 mg $ml^{-1}$ in PBS. In some experiments, tumour perfusion was blocked after drug administration using a D-shaped clamp (Bremner *et al.*, 1990). At subsequent times, mice were sacrificed and tumours rapidly excised and placed in ice-cold PBS. A single-cell suspension was prepared from the entire tumour by mincing the tissue and incubating for 30 min with a mixture of trypsin, collagenase and DNAse as described previously (Olive, 1989).

Mice were irradiated whole body in Plexiglas chambers at a dose rate of 4.5 Gy $min^{-1}$. Following irradiation, mice were sacrificed by cervical dislocation and tumours rapidly excised and placed in ice-cold PBS. A single-cell suspension was prepared from the entire tumour by mincing the tissue and filtering the suspension through 50 μm nylon mesh. The suspension was then centrifuged and resuspended in complete medium.

*Hoechst 33342 tumour cell sorting*

To examine the response of tumour cells to radiation as a function of their distance from the functional vasculature, mice were injected intravenously with 0.1 ml of the fluorescent perfusion stain Hoechst 33342 (8 mg $ml^{-1}$ in PBS) approximately 20 min before asphyxiation or irradiation. After irradiation, mice were sacrificed and tumours were removed and disaggregated enzymatically as previously described (Olive, 1989). Tumour cells were exposed for 5 min on ice to a 1:100 dilution of fluorescein isothiocyanate (FITC)-conjugated goat anti-mouse IgG (Sigma) to stain macrophages which are the most abundant normal cell constituent of this tumour (Olive, 1989). IgG-negative cells were sorted on the basis of Hoechst 33342 concentration into the 10% most dimly fluorescent and 10% most brightly fluorescent populations, using a Becton-Dickinson FACS440 cell sorter (Chaplin *et al.*, 1985).

*DNA damage measured using the comet assay*

For the alkaline comet assay, single cells from spheroids or SCCVII tumours were suspended in ice-cold PBS at a concentration of $2-4 \times 10^4$ cells $ml^{-1}$. Then 0.5 ml of cell suspension ($10^4$ cells) was placed in a 5 ml disposable tube and 1.5 ml of 1% low gelling temperature agarose (Sigma type VII prepared in distilled water and held at 40°C), was added to the tube. Then 1.5 ml was quickly pipetted onto a half-frosted microscope slide and allowed to gel for about 1 min on a cold surface. Slides were carefully submersed in an alkaline lysis solution containing 1 M sodium chloride, 0.03 M sodium hydroxide and 0.1% sarkosyl for 1 h followed by a 1 h wash in 0.03 M sodium hydroxide, 2 mM EDTA, before electrophoresis in a fresh solution of 0.03 M sodium hydroxide, 2 mM EDTA, at 0.6 V $cm^{-1}$ for 25 min. Slides were rinsed and stained for 10 min in 2.5 μg $ml^{-1}$ propidium iodide.

For the neutral comet assay, cells were embedded in agarose as above, then slides were immersed for 4 h at 50°C in 0.5% sodium dodecyl sulphate (SDS), 30 mM EDTA,

**Figure 1** Radiation-induced DNA damage in SCCVII tumours measured using the alkaline comet assay. Mice bearing SCCVII tumours were injected i.v. with Hoechst 33342 and 20 min later were given 30 Gy irradiation while breathing air (**a**–**c**) or after asphyxiation (**d**–**f**). Tumours were rapidly removed, and a single-cell suspension was prepared using mechanical and enzymatic methods. Cells were sorted on the basis of the Hoechst 33342 fluorescence gradient into the 10% most dimly fluorescent cells (**a** and **d**), the 10% most brightly fluorescent cells (**b** and **e**) or the unsorted population (**c** and **f**). Sorted cells were analysed for DNA damage using the alkaline comet assay. A curve-fitting program was used to calculate the hypoxic fraction (HF) in each sample from the air-breathing animal.

pH 8.3. After lysis, some slides were incubated with 0.5 mg ml$^{-1}$ proteinase K (Boerhinger) overnight at 37°C. After lysis, slides were rinsed overnight in Tris–borate–EDTA buffer (Olive *et al.*, 1991). Horizontal gel electrophoresis at 0.6 V cm$^{-1}$ for 25 min was used to separate damaged from undamaged DNA.

Individual cells or 'comets' were viewed using a Zeiss epifluorescence microscope attached to an intensified solid-state charge-coupled device (CCD) camera and image analysis system. For viewing propidium iodide fluorescence, slides were illuminated with green light from a 100 W mercury source using a 580 nm reflector and 590 nm barrier filter. Individual comets were viewed using a 25 × objective and images were analysed using a fluorescence image processing system previously described (Olive *et al.*, 1990). As the number of DNA strand breaks increased, the amount of DNA able to migrate away from the comet head increased proportionate to dose. The 'tail moment', defined as the product of the percentage of DNA in the comet tail multiplied by the distance between the means of the head and tail distributions, and 'DNA content', defined as the total fluorescence associated with an image, were the most informative features (Olive *et al.*, 1990). Tail moment histograms generated from 200 or more comets were subjected to a curve-smoothing algorithm.

### Results

Our previous results have shown that tumour cells from mice breathing air during irradiation show significantly more DNA damage than cells from mice asphyxiated prior to irradiation, and that two populations of cells can be identified on the basis of DNA strand breaks in the cells obtained from the irradiated air-breathing animal (Olive *et al.*, 1992, 1994*a*; Olive, 1994). In Figure 1, tumour cells were sorted on the basis of the Hoechst 33342 fluorescence gradient to provide samples of the most dimly fluorescent 10% of the population (Figure 1a and d) and the most brightly fluorescent 10% of the population (Figure 1b and e). Presumably, those cells close to the functional vasculature at the time of irradiation, i.e. the most brightly fluorescent cells, should be better oxygenated and therefore exhibit a higher proportion of cells displaying an aerobic response. A curve-fitting algorithm was used to calculate the fraction of hypoxic cells in the tumour of the air-breathing mouse (Olive and Durand, 1992). Representative results shown in Figure 1a and b indicate that there is enrichment for hypoxic cells in dimly versus brightly fluorescent populations. Figure 1c shows the average response of the unseparated cells from the

**Figure 2** Tirapazamine-induced DNA damage to cells of Chinese hamster V79 spheroids. Twelve day old spheroids were incubated for 1 h at 37°C with 30 μM tirapazamine in medium equilibrated with 21%, or 0% nitrogen or 10% oxygen. Single cells were analysed for DNA damage and DNA content using the alkaline comet assay. The bivariate display in (**a**) shows results for individual comets from spheroids incubated with tirapazamine under 10% oxygen gassing conditions. Histograms in (**b**–**d**) show representative results for 200 comets.

**Figure 3** Comparison between the alkaline and neutral comet assays for detecting single- and double-strand breaks by tirapazamine and X-rays. (**a**) Chinese hamster V79 spheroids were incubated for 1 h under anoxic conditions with tirapazamine, then analysed for DNA damage using the alkaline (O) or netural (●) version of the comet assay. Samples of cells were also exposed overnight to proteinase K prior to neutral electrophoresis (▼). The means (s.d.) for 100 comets are shown. (**b**) Plateau phase V79 cells were irradiated on ice prior to lysis under alkaline (O) or neutral (●) conditions in the absence of proteinase K. Results show the means (s.d.) for three independent experiments for selected doses.

same tumour. A smaller average tail moment, indicative of less DNA damage, is observed in the tumour of a mouse asphyxiated prior to irradiation compared with the tumour from the air-breathing mouse (Figure 1f). In tumours from animals asphyxiated prior to irradiation, the patterns from dimly (Figure 1d) and brightly (Figure 1e) fluorescent populations are identical, and overall DNA damage is reduced by more than a factor of 2 compared with tumours from air-breathing mice.

Tirapazamine 30 μM produced 20–40 times more DNA single-strand breaks in cells from anoxic spheroids than in cells from aerobic V79 spheroids (Figure 2b and c), which is very different than the 2- to 3-fold differential in radiation damage between aerobic and anoxic cells shown in Figure 1. Heterogeneity in response of the cells from anoxic spheroids was relatively small, indicating that DNA of all cells was damaged to a similar extent. In spheroids equilibrated with 10% oxygen and containing approximately 50% hypoxic cells, the pattern of damage was very broad, encompassing spheroids with tail moments of 0.5–55 (Figure 2a and d). Cells with tail moments similar to those of the fully anoxic spheroids constituted about 10% of the spheroid (Figure 2d).

Previous results have indicated that tirapazamine causes both DNA single- and double-strand breaks (Zeman and Brown, 1989). Using both the alkaline and neutral comet assays, the presence of DNA single- and double-strand breaks was compared following exposure of Chinese hamster V79 spheroids, under anoxia, to tirapazamine. In the absence of proteinase K, there was a small increase in damage at low doses of tirapazamine, but a subsequent decrease at higher doses. Following incubation of slides with 0.5 mg $ml^{-1}$ proteinase K, the amount of DNA damage increased significantly, indicating the presence of protein-linked strand breaks (Figure 3a). The ratio of single- to double-strand breaks is estimated to be about 10 from these data compared with 18 for the X-ray results shown in Figure 3b. Exposure to 100 μM tirapazamine under anoxic conditions produced double-strand breaks equivalent to about 150 Gy.

Tirapazamine induced dose-dependent DNA damage in cells from SCCVII murine tumours (Figure 4a). However, heterogeneity in DNA damage in the tumour observed 30 min after drug administration was relatively small and there was no indication of the presence of a separate drug-resistant population (Figure 4c). Waiting longer between drug injection and tumour removal allowed some of the damage to be repaired (Figure 4b). The range of tail moments increased significantly at 60 and 120 min post injection (Figure 4d and e), allowing resolution of a less damaged population. However, by 4 h after injection, insufficient damage remained to resolve a heavily damaged hypoxic population (Figure 4f).

Fluorescence-activated cell sorting experiments using the perfusion probe Hoechst 33342 were conducted to determine whether the heavily damaged population of cells was in fact hypoxic. Results indicate that there was a significant difference in the responses for the brightly (well-perfused) and dimly (poorly perfused) fluorescent cells removed from tumours 60 and 120 min after injection of 20 mg $kg^{-1}$ tirapazamine. However, a difference between sorted populations was not observed at early times after injection (Figure 5).

Clamping tumours for 1 h following i.p. injection of 20 mg $kg^{-1}$ tirapazamine resulted in a homogeneous pattern of damage when the tumour cells were subsequently analysed for strand breaks using the alkaline comet assay (Figure 6). This result indicates that all of the cells of the tumour are capable of activating and being damaged by tirapazamine. Moreover, in spite of a high cell density, all cells showed similar levels of damage indicating that the available drug had good access to all cells. Results from a mouse breathing air at the time of irradiation are shown for comparison, with the fraction of 'hypoxic' cells equated arbitrarily to the fraction of cells with tail moments $>24$. The basis for this choice was that the SCCVII tumour displays about 20% hypoxic cells measured using the comet assay (Olive and Durand, 1992). The enrichment for hypoxic cells in the dimly fluorescent population seems more convincing for tirapazamine than for X-rays. The SCCVII tumour undergoes transient changes in perfusion which can prevent accurate selection of radiation-resistant hypoxic cells using the Hoechst 33342 cell sorting method (Chaplin *et al.*, 1987). Perhaps the 1–2 h contact time of the drug with cells of this tumour provides

**Figure 4** Tirapazamine-induced DNA damage to SCCVII tumours growing in C3H mice. (**a**) Animals were injected i.p. with different amounts of tirapazamine 30 min prior to tumour excision and preparation of single-cell suspensions. The means (s.d.) for three experiments are shown for selected doses. (**b**) Mice were injected with 20 mg $kg^{-1}$ tirapazamine, then tumours were removed at various times after injection and analysed for DNA damage using the alkaline comet assay. The means (s.d.) for three tumours are shown for selected time points. (**c**–**f**) Representative histograms for four sample times following 20 mg $kg^{-1}$.

**Figure 5** Differential damage to poorly *vs* well-perfused SCCVII tumour cells. Mice were injected i.p. with 20 mg $kg^{-1}$ tirapazamine prior to i.v. injection with Hoechst 33342. Tumours were excised 5 min later and a single-cell suspension was prepared. The means (s.d.) for three separate experiments are shown. In (**b**) and (**c**), representative histograms from 200 comets are shown for dimly fluorescent (**b**, ▽) and brightly fluorescent (**c**, ●) tumour cells examined 60 min after tirapazamine injection.

adequate time to damage all of the hypoxic cells, even those which are aerobic for some period of time during this interval.

The disappearance of DNA damage from cells of tumours shown in Figure 4b is indicative of strand break rejoining. Using SCCVII tumour cells *in vitro*, the rate of strand break rejoining was measured following a 1 h incubation of the cells with 200 μM tirapazamine under aerobic conditions, with repair also observed under aerobic conditions (Figure 7). The kinetics was essentially exponential with a half-time of about 50 min. V79 spheroids were incubated for 1 h with 10 μM tirapazamine under nitrogen, or with 200 μM tirapazamine under aerobic conditions. Damaged spheroids were then allowed to repair under aerobic or anoxic conditions. Whether damage was induced under aerobic or hypoxic conditions, rejoining of breaks occurred with a half-time of about 1 h when cells were subsequently incubated under air (Figure 8a and b, closed symbols). However, the rate of strand break rejoining was inhibited by incubation of cells under anoxic conditions, and the half-time of repair increased to about 2 h (Figure 8a and b, open symbols). The distribution of damage shown in Figure 8c–f indicates that most cells of the population repaired damage with similar kinetics. Therefore, the slower rejoining kinetics under nitrogen was not the result of the presence of a small population of cells undergoing DNA degradation.

### Discussion

The ability of tirapazamine to preferentially damage the DNA of hypoxic cells can be readily demonstrated in both V79 spheroids and SCCVII murine tumours. The differential between the response of fully oxic and fully anoxic cells is considerably larger than the differential for ionising radiation-induced DNA damage. The enrichment for hypoxic cells seen in the dimly fluorescent populations of tumour cells is more convincing for tirapazamine (Figures 5 and 6) than for X-rays (Figure 1). The application of this drug should therefore improve the ability of the comet assay to resolve small fractions of hypoxic cells in solid tumours. However, before tirapazamine damage to DNA can be used as a reliable indicator of radiobiological hypoxia, several factors must be considered. The number of strand breaks observed in cells from tirapazamine-treated tumours is the sum of the damage induced by that point in time and the repair that has occurred prior to tumour excision. Our results with spheroids indicate a difference in strand break rejoining rate for spheroids incubated under aerobic or anoxic conditions, so that we cannot assume that rejoining rate (and thus ultimate damage) is independent of oxygenation and other factors. Damage is also a function of the bioreductase activity of the tumour cell, which may vary within a tumour as a result of genetic or environmental influences. Since the distinction between aerobic and hypoxic cells is made only on the basis of DNA strand breaks, it is necessary to assume that bioreductase activity does not vary within the tumour. Our results with tumours from asphyxiated mice (Figure 6) support this position, but again this assumption may not always be valid. The relation between tirapazamine-induced DNA damage and oxygen tension is not likely to be identical to the relation between radiosensitivity and oxygen tension (Koch, 1993), adding another complication to interpretation of comet histograms. It should be noted that none of these problems applies to the use of ionising radiation-induced DNA damage to detect hypoxic cells. Moreover, comet histograms following irradiation can generally be fit to a two-component curve which accurately identifies aerobic and radiobiologically hypoxic cells (Figure 1). Results obtained with tirapazamine are perhaps more analogous to oxygen electrode histograms, in which a somewhat arbitrary distinction must be made between aerobic and radiobiologically hypoxic tissue on the basis of oxygen tension measurements.

The ability to discriminate damaged from undamaged cells 1–2 h after tirapazamine injection but not 20–30 min after injection could be due to several factors. A possible explanation for the reduced heterogeneity observed in tumour cells at early times after drug injection (Figure 4b) is that hypoxia is induced in the tumour cells during the process of tumour excision, thus allowing more cells to metabolise the drug when plasma levels of tirapazamine were still high; the plasma half-life of tirapazamine in mice is 15–30 min (Minchinton *et al*., 1992; Walton and Workman, 1993). Care was taken to minimise metabolism of the drug by rapid excision and cooling in ice-cold buffer, and no difference in the amount of cell killing was observed in tumour cells removed 30 min *vs* 2 h after tirapazamine injection (PL Olive, unpublished results). Of course, DNA damage measured at any point in time is an indication of both induction and repair. Poorly perfused cells, which undergo more damage as a result of their hypoxic status, may continue to be damaged

**Figure 6** Influence of tumour clamping on tirapazamine-induced DNA damage in cells sorted on the basis of Hoechst 33342 fluorescence. (**a**–**c**) Mice were injected with 20 mg $kg^{-1}$ tirapazamine and 60 min later with Hoechst 33342 followed 5 min later by sacrifice and preparation of a single-cell suspension for cell sorting and analysis using the alkaline comet assay. (**d**–**f**) Mice were injected with 20 mg $kg^{-1}$ tirapazamine followed 30 min later by clamping of the tumour to produce hypoxia. After 60 min, the clamp was removed for 5 min, then the mouse was injected i.v. with Hoechst 33342 followed 5 min later by sacrifice and preparation of a single-cell suspension. (**a** and **d**) The response of the dimmest 10% of cells. (**b** and **e**) The brightest 10%. (**c** and **f**) The unsorted population.

**Figure 7** Rejoining of tirapazamine-induced strand breaks in SCCVII tumour cells *in vitro*. Single cells were exposed to 200 μM tirapazamine under aerobic conditions followed by extensive washing and incubation in fresh medium under aerobic conditions. Cells were analysed for DNA damage using the alkali comet assay. The mean (s.d.) for 100 comets per time point is shown.

by small concentrations of circulating drug which fail to affect better oxygenated cells. The spheroid results shown in Figure 8 indicate another possibility. Cells maintained under hypoxic conditions after treatment with tirapazamine show a decreased rate of strand break rejoining, perhaps as a result of the decreased energy status of hypoxic cells. An increase in heterogeneity in DNA damage with time after treatment would occur as hypoxic cells, which are already more heavily damaged, fail to rejoin breaks as rapidly as aerobic cells.

While we have previously shown that only the outer cell layers of spheroids incubated with 25 μg $ml^{-1}$ tirapazamine under anoxic conditions are sensitive to the drug, presumably as a result of rapid drug consumption by external cells (Durand and Olive, 1992), the response in terms of DNA damage is relatively homogeneous, that is, all the cells of the anoxic spheroids show strand breakage (Figure 2c). Similarly, when tumours are clamped after tirapazamine injection, all of the cells show the same amount of DNA damage, regardless of their position relative to the vasculature (Figure 6). The apparent lack of a correlation between DNA damage and cell killing is reminiscent of effects of the topoisomerase II poison, etoposide (Olive *et al*., 1993*b*). Approximately ten single-strand breaks are produced for each (protein-linked) double-strand break (Figure 3), a ratio about 2-fold lower than observed for ionising radiation but similar to that observed for etoposide. Protein-linked breaks are not observed using ionising radiation since no additional sensitivity is obtained in the neutral comet assay by including proteinase K during the lysis procedure following irradiation (Olive *et al*., 1992), but proteinase K was found to improve detection of double-strand breaks produced by etoposide (Olive and Banáth, 1993*a*).

Another interesting observation is that the kinetics of strand break rejoining in SCCVII cells appears to be exponential (Figures 7 and 8), which could indicate that only one type of lesion is being repaired. Rejoining of breaks is much slower than observed for radiation-induced single-strand breaks, which has been shown to be 3–5 min using this assay (Olive and Banáth, 1993*b*). Even rejoining of radiation-induced double-strand breaks, measured using the comet assay, occurs with a rapid half-time of about 15 min (Olive *et al*., 1994*b*), although a slower component displays a half-time of 1 h or more. Biederman *et al*. (1991) reported a half-time of double-strand break rejoining of about 0.75 h for CHO cells incubated under anoxic conditions with 25 μM tirapazamine, similar to the kinetics for single-strand break rejoining shown in Figure 7 and 8. In the model for tirapazamine toxicity proposed by Brown (1993), lesions produced by this drug are more difficult to repair, analogous to local multiply damaged sites proposed by Ward (1981) for ionising radiation. The slow strand break rejoining kinetics is consistent

**Figure 8** Rejoining of tirapazamine-induced strand breaks in Chinese hamster V79 spheroids. Spheroids were incubated for 1 h with either 10 μM tirapazamine under nitrogen gassing conditions (**a**) or 200 μM under aerobic conditions (**b**). Following extensive washing, intact spheroids were allowed to repair damage under either aerobic conditions (●) or anoxic conditions (○). Single cells were prepared by trypsin treatment and analysed for DNA damage. (**c**–**f**) Representative histograms for spheroids incubated under anoxic conditions and allowed to repair damage for 1 h under nitrogen (**c**) or air (**d**) or spheroids incubated under air and allowed to repair damage for 1 h under nitrogen (**e**) or air (**f**).

with such a model, and may also indicate that all lesions created by this drug (even single-strand breaks) are more difficult to handle by DNA repair systems.

In summary, tirapazamine produced 20–40 times more DNA strand breaks in anoxic than in aerobic cells. In multicell systems such as spheroids and tumours, tirapazamine damage measured in individual cells reflects the heterogeneity in oxygen content. While unambiguous identification of radiobiologically hypoxic cells is probably not possible with this approach, measurement of DNA damage to individual cells provides an indication of the range and distribution of cellular oxygenations. This information may be useful in estimating tumour and tissue hypoxia.

**Acknowledgements**
This work was supported by Grant Number CA 37879 awarded by the National Cancer Institute DHHS. The author acknowledges the expert technical assistance of Charlene M Vikse. Dr J Martin Brown generously supplied the tirapazamine and provided helpful insights during the course of this study.

**References**

BIEDERMANN KA, WANG J, GRAHAM RP AND BROWN JM. (1991). SR 4233 cytotoxicity and metabolism in DNA repair-competent and repair deficient cell cultures. *Br. J. Cancer*, **63**, 358–362.

BREMNER JCM, STRATFORD IJ, BOWLER J AND ADAMS GE. (1990). Bioreductive drugs and the selective induction of tumor hypoxia. *Br. J. Cancer*, **61**, 717–721.

BROWN JM. (1993). SR4233 (tirapazamine): a new anti-cancer drug exploiting hypoxia in solid tumours. *Br. J. Cancer*, **67**, 1163–1167.

BUSH RS, JENKIN RDT, ALLT W AND BEALE FA. (1978). Definitive evidence for hypoxic cells influencing cure in cancer therapy. *Br. J. Cancer*, **37**, 302–306.

CAHILL A AND WHITE INH. (1990). Reductive metabolism of 3-amino-1,2,4-benzotriazine-1,4-dioxide (TIRAPAZAMINE) and the induction of unscheduled DNA synthesis in rat and human derived cell lines. *Carcinogenesis*, **11**, 1407–1411.

CHAPLIN DJ, OLIVE PL AND DURAND RE. (1987). Intermittent blood flow in a murine tumor: radiobiological effects. *Cancer Res.*, **47**, 597–601.

CHAPMAN DJ, DUGLE DL, REUVERS AP, MEEKER BE AND BORSA J. (1974). Studies on the radiosensitizing effect of oxygen in Chinese hamster cells. *Int. J. Radiat. Biol.*, **26**, 383–389.

DURAND RE AND OLIVE PL. (1992). Evaluation of bioreductive drugs in multicell systems. *Int. J. Radiat. Oncol. Biol. Phys.*, **22**, 689–692.

KOCH CJ. (1993). Unusual oxygen concentration dependence of toxicity of SR-4233, a hypoxic cell toxin. *Cancer Res.*, **53**, 3992–3997.

LADEROUTE K, WARDMAN P AND RAUTH AM. (1988). Molecular mechanisms for the hypoxia-dependent activation of 3-amino-1,2-4-benzotriazine 1,4-dioxide (SR 4233). *Biochem. Pharmacol.*, **37**, 1487–1495.

MINCHINTON AI, LEMON MJ, TRACY M, POLLART D, MARTINEZ A, TOSTO L AND BROWN JM. (1992). Second generation 1,2,4-benzotriazine 1,4-di-N-oxide bioreductive anti-tumour agents: pharmacology and activity in vitro and in vivo. *Int. J. Radiat. Oncol. Biol. Phys.*, **22**, 701–705.

OKUNIEFF P, HOECKEL M, DUNPHY EP, SCHLENGER K, KNOOP C AND VAUPEL P. (1993). Oxygen tension distributions are sufficient to explain the local response of human breast tumors treated with radiation alone. *Int. J. Radiat. Oncol. Biol. Phys.*, **26**, 631–636.

OLIVE PL. (1989). Distribution, oxygenation and clonogenicity of macrophages in a murine tumor. *Cancer Commun.*, **1**, 93–100.

OLIVE PL. (1994). Radiation-induced reoxygenation in the SCCVII murine tumor: evidence for a decrease in oxygen consumption and an increase in tumor perfusion. *Radiother. Oncol.*, **32**, 37-46.

OLIVE PL AND DURAND RE. (1992). Detecting hypoxic cells in a murine tumor using the comet assay. *J. Natl Cancer Inst.*, **85**, 707-711.

OLIVE PL AND BANÁTH JP. (1993*a*). Detection of DNA double-strand breaks through the cell cycle after exposure to X-rays, bleomycin, etoposide and $^{125}$IdUrd. *Int. J. Radiat. Biol.*, **64**, 349-358.

OLIVE PL AND BANÁTH JP. (1993b). Induction and rejoining of radiation-induced DNA single-strand breaks: tail moment as a function of position in the cell cycle. *Mutat. Res.*, **294**, 275-283.

OLIVE PL, BANÁTH JP AND DURAND RE. (1990). Heterogeneity in radiation-induced DNA damage and repair in tumor and normal cells measured using the comet assay. *Radiat. Res.*, **122**, 86-94.

OLIVE PL, WLODEK D AND BANÁTH JP. (1991). DNA double-strand breaks measured in individual cells subjected to gel electrophoresis. *Cancer Res.*, **51**, 4671-4676.

OLIVE PL, WLODEK D, DURAND RE AND BANÁTH JP. (1992). Factors influencing DNA migration from individual cells subjected to gel electrophoresis. *Exp. Cell Res.*, **198**, 259-267.

OLIVE PL, DURAND RE, LE RICHE J, OLIVOTTO I AND JACKSON SM. (1993*a*). Gel electrophoresis of individual cells to quantify hypoxic fraction in human breast cancers. *Cancer Res.*, **53**, 733-736.

OLIVE PL, BANÁTH JP AND EVANS HH. (1993*b*). Cell killing and DNA damage by etoposide in Chinese hamster V79 monolayers and spheroids: influence of growth kinetics, growth environment and DNA packaging. *Br. J. Cancer*, **67**, 522-530.

OLIVE PL, VIKSE CM AND DURAND RE. (1994*a*). Hypoxic fractions measured in murine tumors and normal tissues using the comet assay. *Int. J. Radiat. Oncol. Biol. Phys.*, **29**, 487-491.

OLIVE PL, MACPHAIL SH AND BANÁTH JP. (1994*b*). Lack of correlation between DNA double-strand break induction/rejoining and radiosensitivity in six human tumor cell lines. *Cancer Res.*, **54**, 3939-3946.

ÖSTLING O AND JOHANSON KJ. (1984). Microelectrophoretic study of radiation-induced DNA damages in individual mammalian cells. *Biochem. Biophys. Res. Commun.*, **123**, 291-298.

STONE HB, BROWN JM, PHILLIPS TL AND SUTHERLAND RM. (1993). Oxygen in human tumors: correlations between methods of measurement and response to therapy. *Radiat. Res.*, **136**, 422-434.

WALTON MI AND WORKMAN P. (1993). Pharmacokinetics and bioreductive metabolism of the novel benzotriazine di-N-oxide hypoxic cell cytotoxin WIN 59075 (SR 4233: NCS 130181) in mice. *J. Pharmacol. Exp. Ther.*, **265**, 938-947.

WANG J, BIEDERMAN KA AND BROWN JM. (1992). Repair of DNA and chromosome breaks in cells exposed to SR 4233 under hypoxia or to ionizing radiation. *Cancer Res.*, **52**, 4473-4477.

WANG J, BIEDERMANN KA, WOLF CR AND BROWN JM. (1993). Metabolism of the bioreductive cytotoxin SR 4233 by tumor cells: enzymatic studies. *Br. J. Cancer*, **67**, 321-325.

WARD JF. (1981). Some biochemical consequences of the spatial distribution of ionizing radiation-produced free radicals. *Radiat. Res.*, **86**, 185-195.

ZEMAN EM AND BROWN JM. (1989). Pre- and post-irradiation radiosensitization by SR 4233. *Int. J. Radiat. Oncol. Biol. Phys.*, **16**, 967-971.

ZEMAN EM, BROWN JM, LEMON MJ, HIRST VK AND LEE WW. (1986). SR 4233: a new bioreductive agent with high selective toxicity for mammalian cells. *Int. J. Radiat. Oncol. Biol. Phys.*, **12**, 1239-1241.